# The validity of footprint-based measures of arch structure: revisiting the debate of fat versus flat feet in adults

**DOI:** 10.1186/1757-1146-5-S1-O54

**Published:** 2012-04-10

**Authors:** Hin-Chung Lau, Scott C Wearing, Nicole L Grigg, James E Smeathers

**Affiliations:** 1 Faculty of Health Sciences and Medicine, Bond University, Queensland, 4229, Australia; 2 Centre of Excellence for Applied Sport Science Research, Queensland Academy of Sport, Queensland, 4111, Australia; 3 Institute of Health and Biomedical Innovation, Queensland University of Technology, Queensland, 4059, Australia

## Background

Previous research employing footprint-based measures of arch structure, such as the arch index (AI), have indicated that obesity results in a ‘flatter’ foot type [1]. In the absence of radiographic measures, however, definitive conclusions regarding the osseous alignment of the foot cannot be made. This study evaluated the effect of Body Mass Index (BMI) on radiographic and footprint-based measures of adult arch structure.

## Materials and Methods

**Figure 1:**
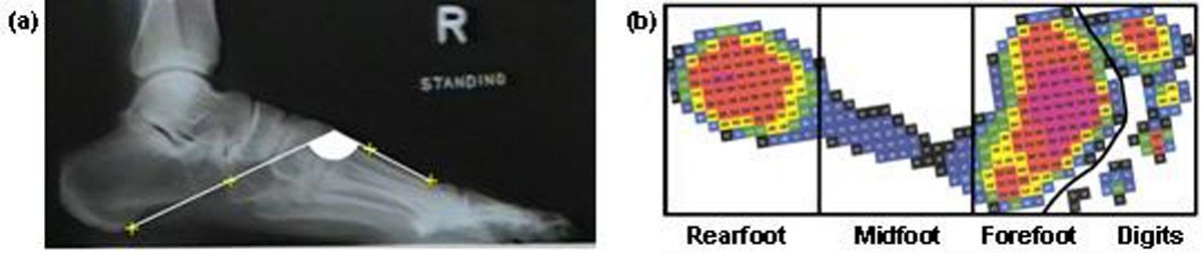
Illustration of radiographic (a), and footprint-based measures of arch structure (b).

A convenience sample of 30 healthy adults (10 male and 20 female, mean (±SD) age 47.9 ± 11.6 years, height 1.68 ± 0.1m, body weight 80.8 ± 10.2kg, BMI 28.8 ± 2.9kg.m^-2^) were recruited. The calcaneal-first metatarsal angle (CMT1) (Figure 1a) was derived from weight-bearing lateral radiographs [2], while the AI was calculated from electronic footprints (EMED-SF, Novel GmbH, Germany) as the ratio of the area of the midfoot relative to the total foot contact area ignoring the digits (Figure 1b). Multiple regression models were used to evaluate the independent influence of BMI, age, and arch structure (as defined by CMT1 angle) on the footprint-based AI, and investigate whether BMI, age, and AI were significant predictors of CMT1 angle.

## Results

Both BMI (β=0.39, P=0.04) and CMT1 angle (β=0.51, P<0.01) were significant predictors of footprint-based measures of arch structure (AI). The CMT1 angle accounted for 30% of the variability in AI, while BMI accounted for 15% of the variation in AI. In contrast, CMT1 angle was not significantly associated with BMI (β=-0.03, P=0.89) when AI and age were held constant. Age was not a significant predictor of either index.

## Conclusions

Adult obesity does not influence the osseous alignment of the medial longitudinal arch, but selectively distorts footprint-based measures of arch structure. Consequently, footprint-based measures should be interpreted with caution when comparing groups of adults with varying body composition.
